# Supplementary material for: The feasibility, appropriateness, and usability of mobile neuro clinics in addressing the neurosurgical and neurological demand in Uganda
Source: PLoS One. 2024 Jun 24;19(6):e0305382. doi: 10.1371/journal.pone.0305382 (PMC11195962; doi:10.1371/journal.pone.0305382)
Supplement: S1 File — (DOCX) [file pone.0305382.s001.docx]

## **S5 Interview Guides**

## **MHC Providers Interview Guide:**

**MHC provider interview guide**

Mobile Health Clinics – Interview Guide

**Description:**

This is the cover sheet for the interview guide for the study (using mobile clinics to deliver neurosurgery and neurology care to populations living in remote and rural communities of Uganda). This is the copy for mobile health care providers.

**Introduction**

Hello, my name is _________ Today, I want to talk to you about your perspectives on implementing Mobile Health Clinics to deliver neurosurgery and neurology care to remote and rural populations of Uganda as a part of this research study. I will be asking questions about your perspectives on neurosurgical and neurological healthcare, Mobile Health Clinics, and the possibility of integrating both Mobile Health Clinics and neurosurgical and neurological healthcare services. All of your responses are valuable, so please feel free to share whatever you think is important.

Do you have any questions before we begin?

------------------------------------------------------------------------------------------------------------

**INTERVIEW COVER SHEET**

**COMPLETE ONE FOR EACH INTERVIEW**

INTERVIEW NUMBER       ______________________________

INTERVIEWER(S)             ______________________________

INTERVIEW DATE             _______________________________

LANGUAGE OF INTERVIEW:        English

**CONSENT** START TIME    _______________________________

**CONSENT** END TIME                _______________________________

**Length of time for consent process:**  _______________________________

**INTERVIEW** START TIME        _______________________________

**INTERVIEW** END TIME                _______________________________

**Length of time for interview:**  _______________________________

**COMMENTS**

CONSENT:  DESCRIBE ANY COMMENTS ABOUT CONSENT PROCESS

INTERVIEW COVID mitigation procedures:  Describe all steps taken by both interviewer and participants.

INTERVIEW LOCATION:  Describe where the interview took place, and notes about recordings, etc

INTERVIEW NOTES, SPECIAL ISSUES OR PROBLEMS; Add your observations about the interview; also include questions or concerns voiced by the participants.

TRANSCRIPTION: Describe your experience transcribing this interview: did you have any trouble with hearing the participant speak? Was there frequent background noise?  etc

**INFORMANT DEMOGRAPHICS**

**INSTRUCTIONS:  one demographic data sheet for EACH participant**

| Gender |  |
| --- | --- |
| Age |  |
| What role do you serve in your mobile health clinic? |  |
| Do you work full-time or part time?  If part time: Do you have any other jobs |  |
| How long have you worked with your mobile clinic? |  |
| Where does your mobile clinic operate? |  |
| What services does your mobile clinic offer? |  |
| Are you confident in your ability to communicate in English? |  |
| Other |  |

| Parent In-depth Interview Guide | | |
| --- | --- | --- |
| Question | **Focus of Question Related to Methods or study Aims** | **Probes** |
| Part 1- First, I want to get a general sense of your understanding of neurosurgery and neurology care. Just answer these questions as fully as you can and if you’re not sure about a question, please don’t hesitate to ask for clarification. | | |
| Based on the educational material presented on neurosurgery and neurology care and your previous experience, how would you describe your understanding of neurosurgery and neurology care? | To get a better understanding of participant’s background understanding of neurosurgery and neurology |  |
| When were you first introduced to the concept of neurosurgery and neurology? | To build rapport; to ease the participant into talking about experiences |  |
| How many patients do you know could benefit from better access to neurosurgery and neurology services? | To get a better understanding of participant’s personal experiences with the unmet need for neurosurgical and neurological services | Please ensure they say a quantitative value. (i.e, none ≤ ) |
| In your own opinion, do you think Mobile Health Clinics deliver quality, safe and timely health care services to underserved communities? | to assess relative advantage | Why or why not? |
| Part 2-  Now we will switch to discussing Mobile Health Clinics and how they deliver care in Uganda | | |
| In your own words, please describe your current job responsibilities within your Mobile Health Clinic? | to understand background within MHCs | Have you ever worked at another mobile health clinic? |
| What services do you believe patients look for from mobile health clinics? | to assess patient needs and resources | Are you able to offer all of the services patients are looking for at your mobile health clinic? |
| Of the patients that receive care, describe in your own words the shared perceptions patients have on using mobile clinics services? | to assess knowledge and beliefs | What does the community think of using mobile health clinics? |
| What do you consider to be critical to building a high quality Mobile Health Clinic infrastructure? | to assess patient needs and resources | What factors should someone who wants to start a mobile health clinic consider? |
| In your opinion, what are the challenges and barriers mobile health clinics face? | to assess compatibility | What do you believe will help solve these challenges? |
| In your opinion, how difficult was it to integrate/maintain your mobile health clinic into the community you currently serve? | to assess complexity | What aspects made it more difficult to offer services in the community? What aspects made it more convenient? |
| How does your Mobile Health Clinic best quantify reasonable/satisfactory results? | to assess appropriateness |  |
| Part 3 - Now we will discuss using Mobile Health Clinics to deliver Neurosurgery and Neurology care to rural and remote communities of Uganda. Just answer these questions as fully as you can and if you’re not sure about a question, please don’t hesitate to ask for clarification. | | |
| In your opinion, do you believe Mobile Health Clinics can deliver quality, safe, and timely neurosurgical and neurological care? | to assess usability |  |
| How can this benefit populations living in remote and rural communities? |  |  |
| How can this negatively affect the populations living in remote communities? |  |  |
| What do you consider to be the critical needs to build a high-quality Mobile Neuro Clinic infrastructure? | to assess patient needs and resources | What factors should be considered to start such an infrastructure? |
| What challenges do you expect when building a high-quality Mobile Neuro Clinic infrastructure? | to assess complexity | How long would it take to build?  What resources might be the most difficult to get? |
| In your opinion, what are the estimated resources needed to start a Mobile Neuro Clinic?        In your opinion, would you consider working in a mobile health clinic that delivers neurosurgery and neurology care to remote and rural communities?      How do you think rural and remote communities would respond to seeing a Mobile Health Clinic that offers a specialty of neurosurgery and neurology care? | to assess needs and resources and assess design quality and packaging.        to assess implementation climate                to assess culture | Funding? Governmental support? Community support? Staffing? Supplies?        If yes, why and how difficult would it be to integrate serving in mobile health clinics into your workflow?    If not, why and who would you see best fit in such a role?      If negative response: What could help ease a mobile health clinic that offers neurosurgery and neurology care into the rural and remote community?  If positive response: What could help sustain a positive response.  What type of education might help promote Mobile Neuro Clinics? |
| In your opinion, how would one integrate mobile health clinics to deliver neurosurgery and neurology care in Uganda? | to assess implementation climate | What aspects would make it more difficult?  What aspects would make it more convenient? |
| Part 4- Next, we will be talking about the potential barriers the intervention Mobile Neuro Clinics may face and the possible solutions to these potential barriers. Just answer these questions as fully as you can and if you’re not sure about a question, please don’t hesitate to ask for clarification. | | |
| In your opinion, what are the limiting factors of designing mobile neuro clinics? | to assess complexity |  |
| In your opinion, what are the potential barriers to implementing mobile neuro clinics? | to assess compatibility | What solutions have you developed to meet these barriers in your mobile health clinics? |
| In your opinion, what additional concerns/conditions might one have with a mobile clinic that offers only neurosurgery and neurology care instead of generalized care? | To assess relative priority  to assess design quality and packaging | What advice would you offer to someone who wants to run a mobile clinic to deliver only neurosurgery and neurology care? |
| In your opinion, how difficult would it be for the local people to visit a mobile health clinic? | to assess adaptability | In your opinion, what would the local people expect when they visit the mobile neuro clinic? |
| In your opinion, how would implementing mobile neuro clinics be received by other mobile health clinics in Uganda? | to assess relative priority | Tell me more about the relationships between mobile health clinics within Uganda, if any? |
| What is your overall perception of starting mobile clinics that provide neurosurgery and neurology care to people living in remote and rural Uganda? | to assess the relative advantage |  |
| Finally, do you have any questions or anything else you’d like to add?  Thank you for taking the time to speak with us today | | |

## **Neurological care Providers Interview Guide:**

**Neurological care provider interview guide**

Neurosurgeon and Neurology – Interview Guide

**Description:**

This is the cover sheet for the interview guide for the study (using mobile clinics to deliver neurosurgery and neurology care to populations living in remote and rural communities of Uganda). This is the copy for neurosurgery and neurology healthcare providers.

**Introduction:**

Hello, my name is _________ Today, I want to talk to you about your perspectives on implementing Mobile Health Clinics to deliver neurosurgery and neurology care to remote and rural populations of Uganda as a part of this research study. I will be asking questions about your perspectives on neurosurgical and neurological healthcare, Mobile Health Clinics, and the possibility of integrating both Mobile Health Clinics and neurosurgical and neurological healthcare services. All of your responses are valuable, so please feel free to share whatever you think is important.

Do you have any questions before we begin?

------------------------------------------------------------------------------------------------------------

**INTERVIEW COVER SHEET**

**COMPLETE ONE FOR EACH INTERVIEW**

 INTERVIEW NUMBER           ______________________________

INTERVIEWER (S)                   ______________________________

INTERVIEW DATE                  _______________________________

LANGUAGE OF INTERVIEW:  English

**CONSENT** START TIME           ______________________________

**CONSENT** END TIME               ______________________________

**Length of time for consent process:**  ______________________________

**INTERVIEW** START TIME      ______________________________

**INTERVIEW** END TIME         ______________________________

**Length of time for interview:**  ______________________________

**COMMENTS**

CONSENT:  DESCRIBE ANY COMMENTS ABOUT CONSENT PROCESS

INTERVIEW COVID mitigation procedures:  Describe all steps taken by both interviewer and participants.

INTERVIEW LOCATION:  Describe where the interview took place, and notes about recordings, etc

INTERVIEW NOTES, SPECIAL ISSUES OR PROBLEMS; Add your observations about the interview; also include questions or concerns voiced by the participants.

TRANSCRIPTION: Describe your experience transcribing this interview: did you have any trouble with hearing the participant speak? Was there frequent background noise?  Etc

**INFORMANT DEMOGRAPHICS**

**INSTRUCTIONS:  one demographic data sheet for EACH participant**

| Gender |  |
| --- | --- |
| Age |  |
| What hospitals do you work at? |  |
| What do you do for work? |  |
| Do you work full time or part time? |  |
| How long have you worked in your position? |  |
| What title do you hold? |  |
| If resident,  How long have you been a resident? |  |
| Are you confident in your ability to communicate in English? |  |
| Other |  |

| Parent In-depth Interview Guide | | |
| --- | --- | --- |
| Question | **Focus of Question Related to Methods or study Aims** | **Probes** |
| Part 1- First, I want to get a general sense of your understanding of Mobile Health Clinics. Just answer these questions as fully as you can and if you’re not sure about a question, please don’t hesitate to ask for clarification. | | |
| Based on the educational material presented and your previous experience, can you describe Mobile Health Clinics in your own words? | To get a better understanding of participant’s MHCs |  |
| When were you first introduced to the concept of Mobile Health Clinics? | To build rapport; to ease the participant into talking about experiences. | Have you ever served in a Mobile Health Clinic setting? |
| How many patients do you personally know access health services at Mobile Health Clinics? | To get a better understanding of participant’s personal experiences with service delivery of Mobile Health Clinics. | Please ensure they say a quantitative value. (i.e, none ≤) |
| In your own opinion, do you think Mobile Health Clinics can deliver quality, safe and timely health care services to underserved communities? | to assess evidence strength and quality |  |
| Part 2- Now we will switch to discussing Neurosurgery and Neurology Care in the context of Uganda. | | |
| In your own words, please describe your current job responsibilities as a neurosurgeon/neurologist/psychiatrist working in Uganda? | to establish/assess background |  |
|  | | |
| How often do you receive consultations from patients and non-patients about their health status in and/or outside the hospital setting? | to assess patient needs and resources. | Are these consultations always related to neurosurgery and neurology care? |
| From your experience, how do people perceive neurosurgery and neurology care in Uganda at large? | to assess knowledge, beliefs and culture | Do people's perceptions of neurosurgery and neurology differ for those living in rural and remote communities as compared to city/urban Uganda? |
| In your opinion, what needs to be done to improve people’s understanding of neurosurgical and neurological care in Uganda?        In your opinion, what are the challenges and barriers to neurosurgery and neurology care? | to assess adaptability              to assess patient needs and resources | If any, are there any points of neurosurgical and neurological care education that currently exist within Uganda that you are aware of?        What do you believe will help solve these challenges?  (Max of 10 mins to answer this question) |
| In your opinion, how difficult is it to deliver neurosurgical and neurology care to rural and remote communities? | to assess implementation climate | What aspects make it more difficult?  What aspects make it more convenient? |
| Part 3. Now we will discuss using Mobile Health Clinics to deliver Neurosurgery and Neurology care to rural and remote communities of Uganda.  Just answer these questions as fully as you can and if you’re not sure about a question, please don’t hesitate to ask for clarification. | | |
| In your opinion, do you think Mobile Health Clinics could deliver quality, safe and timely neurosurgical and neurology care? | to assess relative advantage |  |
| How can Mobile Health clinics that deliver neurosurgical or neurological care benefit populations living in remote and rural communities? |  |  |
| How can Mobile Health clinics that deliver neurosurgical or neurological care negatively affect the populations living in remote communities? |  |  |
| What do you consider to be critical to building a high-quality Mobile Neuro Clinic infrastructure? | to assess design quality and packaging | What factors should be considered to start such an infrastructure? What infrastructures, that you know of, if any, already exist within Uganda that should be considered when building a high quality MNC? |
| How would you measure reasonable/satisfactory results within the mobile health clinic delivering neurosurgery and neurology care?    In your opinion, would you consider working in a mobile health clinic that delivers neurosurgery and neurology care to remote and rural communities? | to assess appropriateness          to assess implementation climate | If YES, how difficult will it be to integrate serving in mobile health clinics into your current workflow if at all possible?    If NO, why not and who would see best fit in such a role? What would need to change in order for you to consider working in a health clinic that delivers neurosurgery and neurology care to remote and rural communities? |
| How do you think rural and remote communities would respond to seeing a Mobile Health Clinic that offers a specialty of neurosurgery and neurology care? | to assess culture |  |
| What neurosurgical and neurological disorders must be considered while designing a mobile neuro clinic for rural and remote communities? | to assess tension of change | Which disorders have the highest possibility of treatment in an MNC?  Which disorders are moderately possible to treat in an MNC?  Which disorders are not at all possible to treat in an MNC? |
| In your opinion, how difficult would it be to integrate mobile neuro clinics to the already existing avenues of providing care in Uganda | to assess implementation climate | What aspects would make it more difficult?  What aspects would make it more convenient? |
| Would you be willing to operate a mobile neuro clinic? | to assess implementation climate | If yes or no: then ask why |
| Part 4. Next, we will be talking about the potential barriers the intervention Mobile Neurosurgery and Neurology Clinics may face and the possible solutions to these barriers. Just answer these questions as fully as you can and if you’re not sure about a question, please don’t hesitate to ask for clarification. | | |
| In your opinion, what are the limiting factors of designing mobile neuro clinics? | to assess design quality and packaging | What are your suggestions on improving the design of mobile neuro clinics? |
| In your opinion, what are the potential barriers to implementing mobile neuro clinics? | to assess compatibility | What solutions can be developed to meet these barriers? |
| In your opinion, what are the estimated resources needed to start a mobile neuro clinic? | to assess patient needs and resources and assess design quality and packaging | Funding? Governmental support? Community support? Staffing? Supplies? |
| In your opinion, what are the additional concerns/conditions that are only special to neurosurgery and neurology care that can make designing a mobile neuro clinic difficult? | to assess design quality and packaging |  |
| In your opinion, how difficult would it be to implement mobile neuro clinics in rural and remote communities? | to assess implementation of change | If yes, how would it change the way you provide treatment to patients? |
| In your opinion, what would be the effects of implementing mobile neuro clinics to the entirety of neurosurgical and neurological care within Uganda? | to assess complexity |  |
| What is your overall perception of starting mobile neuro clinics that provide neurosurgery and neurology care? | to assess the relative advantage |  |
| Finally, do you have any questions or anything else you’d like to add?  Thank you for taking the time to speak with us today | | |
