# Supplementary figures and images for: The feasibility, appropriateness, and usability of mobile neuro clinics in addressing the neurosurgical and neurological demand in Uganda
Source: PLoS One. 2024 Jun 24;19(6):e0305382. doi: 10.1371/journal.pone.0305382 (PMC11195962; doi:10.1371/journal.pone.0305382)

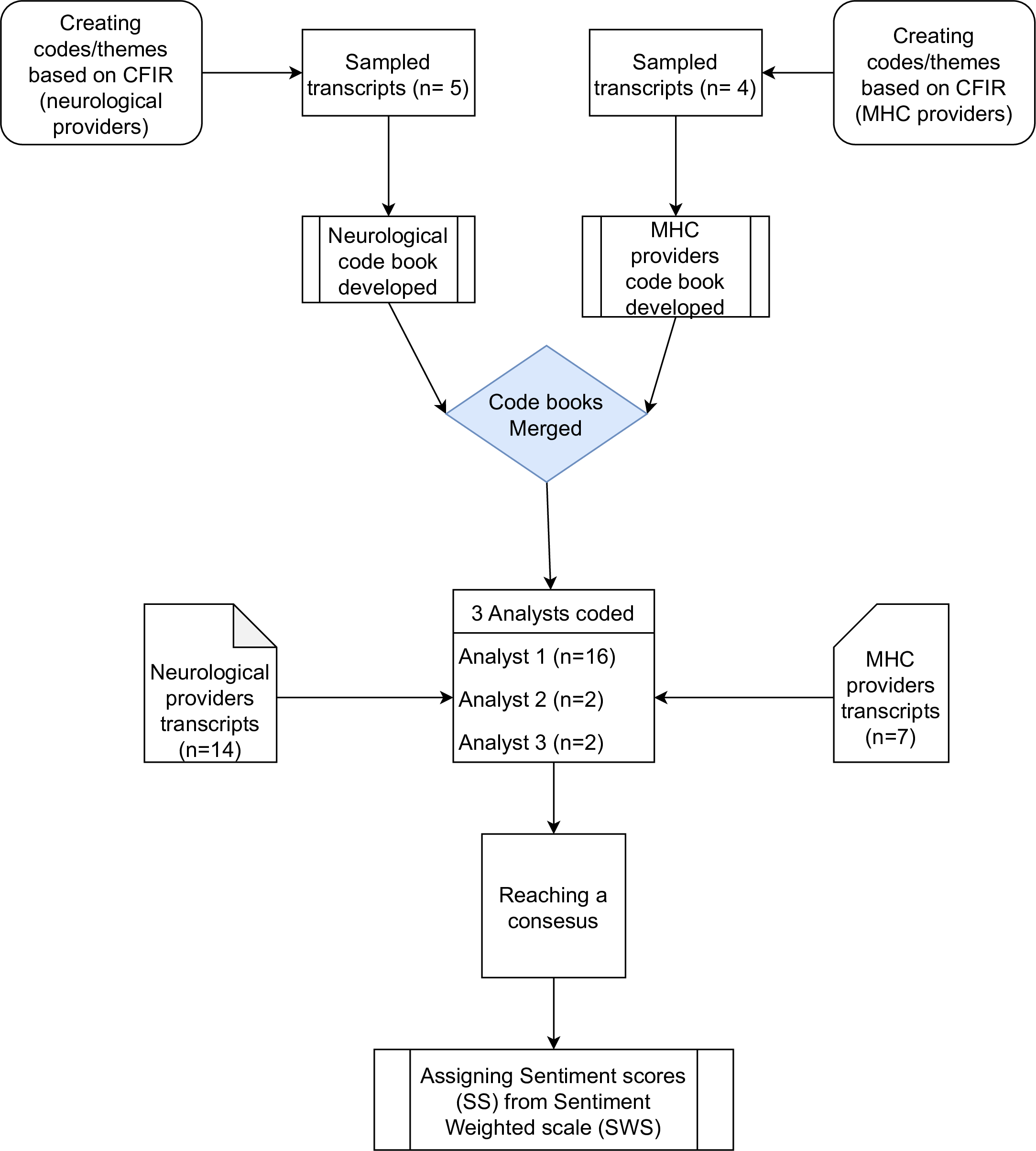

Supplement: S2 File — (TIF) [file pone.0305382.s002.tif]
