# Supplementary material for: The feasibility, appropriateness, and usability of mobile neuro clinics in addressing the neurosurgical and neurological demand in Uganda
Source: PLoS One. 2024 Jun 24;19(6):e0305382. doi: 10.1371/journal.pone.0305382 (PMC11195962; doi:10.1371/journal.pone.0305382)
Supplement: S1 Table — (DOCX) [file pone.0305382.s004.docx]

Supplemental Table 1: Study Constructs

| **TABLE SUMMARIZING STUDY CONSTRUCTS** | |
| --- | --- |
| **Main Study Measure Constructs** | **Definitions** |
| Feasibility | The capability and resulting sample characteristics, possible procedures and outcome measures of MNCs, acceptability of the MNCs and study procedures, resources, and ability to manage and implement the MNCs, and preliminary evaluation of participant responses to MNCs were all assessed. |
| Appropriateness | MNCs are defined as an intervention that will provide cost-effective and equitable neurosurgical and neurological care to people living in Uganda's rural and remote communities. Care providers in Uganda (neurosurgeons, neurologists, and MHC providers) shared their perspectives on the use of MNCs to deliver care to people living in remote and rural areas. |
| Usability | the degree to which MNCs can be utilized by healthcare providers to effectively, efficiently, and satisfactorily provide neurosurgical and neurological care to people living in remote and rural communities in Uganda. |
| **CFIR Constructs** | |
| INNOVATION DOMAIN | Lists all of the characteristics of mobile neuro clinics or mobile health clinics and then goes on to explain how the intervention can be used to meet the demand for local neurological care. |
| OUTER SETTING DOMAIN | The setting in which the inner setting (rural and remote communities) exists: Uganda and it's communities. Collected information describing broad aspects of the country. |
| INNER SETTING DOMAIN | The setting in which the innovation (MNCs) is implemented; rural and remote communities. Data was collected describing rural and remote communities in Uganda. |
| IMPLEMENTATION CLIMATE CONSTRUCTS | Assessed the ability to absorb change, the providers' shared openness to MNCs, and the degree to which MNC use will be rewarded, encouraged, and anticipated. |
| INDIVIDUALS DOMAIN | Collected information about the roles and characteristics of individuals that may be involved in the design and implementation of MNCs. |
| CHARACTERISTICS SUBDOMAIN | documented the roles of providers, their desire to work in MNCs, and current characteristics applicable to the roles in the MNC project. |
| IMPLEMENTATION PROCESS DOMAIN | Assessed the procedures and strategies used to put the innovation into practice. Record the activities and strategies being used to implement the innovation, as well as the implementation process framework. Differentiate between the innovation (the "effects/impact" that persist after implementation is complete) and the implementation process that was used to implement it (activities that end after implementation is complete). |
