# Supplementary material for: The feasibility, appropriateness, and usability of mobile neuro clinics in addressing the neurosurgical and neurological demand in Uganda
Source: PLoS One. 2024 Jun 24;19(6):e0305382. doi: 10.1371/journal.pone.0305382 (PMC11195962; doi:10.1371/journal.pone.0305382)
Supplement: S2 Table — (DOCX) [file pone.0305382.s005.docx]

Supplemental Table 2: CodeBook

| **Parent Code** | **Meaning** | **Child Code** | **Meaning** | **Sub-Child Code** | **Meaning** |
| --- | --- | --- | --- | --- | --- |
| **INNOVATION DOMAIN** | lists all of the characteristics of Mobile Neuro Clinics or Mobile Health Clinics and then goes on to explain how the intervention can be used to meet the demand for local neurological care. | **Adaptability** | describes how well Neurological care can be adapted, tailored, refined, or reinvented to meet local needs in the MHC setting. This implies that the intervention can be adapted from the other contexts; for instance, by utilizing the expertise of the Providers currently operating in Uganda or other regions of the world. |  |  |
|  |  | **Complexity** | Refers to the challenges involved in planning the intervention, from design to implementation to evaluation, etc. | **Centrality** | describes the quality of being essential or vital to MNCs. Its absence may result in poor outcomes, or the process being halted. |
|  |  |  |  | **Disruptiveness** | describes how MNCs could fundamentally alter the practice of neurosurgery and neurology in Uganda. Furthermore, how MNCs might disrupt cultures and communities. |
|  |  |  |  | **Duration** | outlines the anticipated period of time required to develop MNCs and put them into action in order to provide neurological care services to people living in rural and remote communities. |
|  |  |  |  | **Intricacy** | Complicated neurological care details that can make it difficult to fit into an MHC setting. Code the discipline's multidisciplinary nature here; combines several medical disciplines and specializations in an approach to providing neurological care. Also include: Limitations to MHCs/MNCs. |
|  |  |  |  | **Radicalness** | describes aspects that may be out of reach for MNCs. Care providers believe that these aspects are unattainable in MNCs. |
|  |  |  |  | **Scope** | outlines the procedures necessary for creating and putting into effect MNCs. These include all of the MNCs' required pre-design, design-phase, and implementation-phase activities and actions. |
|  |  | **Cost** | estimates the intervention's cost as well as any additional expenses related to designing and putting it into practice (MNCs/MHCs). |  |  |
|  |  | **Design** | describes how the intervention will be packaged, presented, and assembled to better meet the needs of the local community. |  |  |
|  |  | **Relative Advantage** | refers to how healthcare professionals view the benefits of implementing MNCs versus doing nothing at all. | **Perceived Benefits** | Code Quality, safety, Timeliness, and other advantages of MNCs/MHCs here. Quality of MNCs/MHCs: MNCs/MHCs' ability to provide quality neurological care/healthcare to rural and remote communities. Safety of MNCs/MHCs: MNCs' capacity to deliver secure neurological care and healthcare to remote and rural communities. Timeliness of MNCs/MHCs: Capability to provide timely quality neurological care/healthcare to rural and remote communities for improved outcomes. |
|  |  |  |  | **Perceived Concerns** | approach to considering MNCs and MHCs as a means of delivering neurological care. (Both Neutral and Negative perceptions if any) |
| **OUTER SETTING DOMAIN** | The setting in which the Inner Setting exists: Uganda, international communities and any other Communities | **Critical Incidents** | Large-scale and/or unforeseen events obstruct the delivery or implementation of the innovation. | **Medical Error** | Unintended consequences that might come from using MNCs to provide neurological care to communities: include all mistakes that can arise, whether from good or bad practice from MNCs' providers. |
|  |  |  |  | **Other Incidents** | Other incidents that are not medical errors |
|  |  | **Local Attitudes** | Providers' perceptions of how the general public perceives Health care; includes responsibility and convictions about worthiness of the recipients |  |  |
|  |  | **Local Conditions** | conditions that enable the Outer Setting to support the innovation's implementation and/or delivery. | **Demand of neurological care** | explains or estimates the unmet need for neurological care in terms of the patient's demand from the providers' perspective. |
|  |  |  |  | **Economic status** | explains the financial state of the population that MNCs seek to serve |
|  |  |  |  | **Technological conditions** | explains the population's familiarity with technology such as radios, televisions, etc that MNCs seek to serve |
|  |  | **Partnerships & Connections** | External entities such as referral networks, academic affiliations, and professional organization networks are linked to the Inner Setting, which may support MNCs once they are established. | **referral networks** | Describes how patients can be referred through the referral networks or how they are referred through the continuum of care. |
|  |  |  |  | **academic affiliations** | These are organizations/bodies that assist in preparing students for careers in healthcare in Uganda. |
|  |  |  |  | **professional organization networks** | Any networks, local or international, that support healthcare delivery in Uganda |
|  |  | **Policies & Laws** | Legislation, regulations, professional group guidelines and recommendations, or accreditation standards all help to facilitate the implementation and/or delivery of the innovation. |  |  |
|  |  | **External Pressure** | External pressures that are currently driving MHC implementation and/or delivery can also be used to support MNC implementation and/or delivery, or both. | **Societal Pressure: media campaigns/coverage** | Describes how the media can be used to support and raise awareness about MNCs and neurological care. |
|  |  |  |  | **Societal Pressure: advocacy groups** | Support for neurological care/MNC is provided through the use of patient/community voices and opinions on what should be done well in providing neurological care. |
|  |  |  |  | **Societal Pressure: Others** | captures any additional described social movements or demonstrations that may support or impede MNC delivery and/or implementation. |
|  |  |  |  | **Market Pressure** | Avenues that can support or compete with MNCs; compares the likelihood that MNCs will compete favorably or unfavorably with MHCs in Uganda's current market |
|  |  |  |  | **Performance-Measurement Pressure** | Implementation and/or delivery of the innovation are driven by quality standards, benchmarking metrics, or established service goals. |
| **INNER SETTING DOMAIN** | The setting in which the innovation (MNCs) is implemented; rural and remote communities | **Structural Characteristics** | Infrastructure elements that help the innovation to operate properly in Inner Setting. | **Physical Infrastructure** | In the future, concrete, tangible structures that are currently lacking but could be easily created to support neurological care in the hospital setting will help MNCs even more. Concrete, tangible structures that are currently lacking but could easily be created to support neurological care in the country will help MNCs even more in the future. |
|  |  |  |  | **Information Technology Infrastructure** | Data Collection: systems that assist with MHC or current neurological procedures and assist MNCs in data collection |
|  |  |  |  |  | Data Management: systems that assist with MHC or current neurological procedures and assist MNCs in data management |
|  |  |  |  |  | Data analysis and reporting: systems that assist with MHC or current neurological procedures and assist MNCs in data analysis and reporting |
|  |  |  |  | **Workforce Infrastructure** | MHC explains how tasks and responsibilities are organized within and between people and teams, as well as general staffing levels, to support the MHC's functional performance. |
|  |  |  |  |  | Neurological care explains how tasks and responsibilities are organized within and between people and teams, as well as general staffing levels, to support neurological care's functional performance. |
|  |  | **Relational Connections** | High-quality formal and informal networks, teams, and relationships exist both inside and outside of Inner Setting boundaries (e.g., structural, professional). |  |  |
|  |  | **Communications** | Within and outside the boundaries of the Inner Setting, there are excellent formal and informal information sharing practices (e.g., structural, professional). |  |  |
|  |  | **Culture** | shared norms, values, and perspectives throughout the Inner Setting. | **Human Equality-Centeredness** | explains how MNCs promote the inherent equity of the neurological care of all people. |
|  |  |  |  | **Recipient-Centeredness** | explains how MNCs support inherent equity in the provision of neurological care to rural and remote populations. |
|  |  |  |  | **Deliverer-Centeredness** | explains how MNCs support common values, beliefs, and standards regarding providing for, addressing, and caring for the needs of deliverers/providers. |
|  |  |  |  | **Learning-Centeredness** | desire to improve over time and learn more about MNCs, rural and remote communities, and neurosurgery. |
| **IMPLEMENTATION CLIMATE CONSTRUCTS** | the ability to absorb change, the providers' shared openness to MNCs, and the degree to which MNC use will be rewarded, encouraged, and anticipated. | **Tension for Change** | The extent to which providers regard unmet neurosurgery and neurology care needs as intolerable. | **Need for Change** |  |
|  |  |  |  | **No need for change** |  |
|  |  | **Compatibility** | innovation fits with workflows, systems, and processes. | **Compatible aspects** | Aspects that may be perfectly compatible with MNCs |
|  |  |  |  |  | Aspects that are a mix of not being compatible and being compatible: when they both are mentioned |
|  |  |  |  | **Non compatible aspects** | Aspects that may or may not be compatible with MNCs |
|  |  | **Relative Priority** | In comparison to other initiatives, implementing and delivering MNC innovation is critical; The importance of MNC implementation is widely recognized. |  |  |
|  |  | **Incentive Systems** | Incentives and rewards, both tangible and intangible, as well as disincentives and punishments, aid in the implementation and delivery of the innovation. Tangible incentives: tangible motivators for medical staff to provide patients with the best care possible. Intangible incentives: intangible drivers that motivate healthcare professionals to give patients the best care possible. Disincentives: Factors that discourage medical professionals from giving patients the best care possible. Poor mentorship is coded here. |  |  |
|  |  |  |  |  |  |
|  |  |  |  |  |  |
|  |  | **Mission Alignment** | Describes how the innovation's implementation and delivery are consistent with Uganda's overarching commitment, purpose, or goals to improve health for all citizens. |  |  |
|  |  | **Available Resources** | the amount of resources that can be allocated to MNCs and the implementation and ongoing operations of neurological care services. (Most of these only contain the resources that the interviewee specifically mentioned being available). | **Community Support** | Community support, whether positive or negative, for the innovation |
|  |  |  |  | **Governmental Support** | includes all governmental bodies that can participate in the creation and operation of MNCs |
|  |  |  |  | **Other Resources** | Funding: Explains any sources of funds that are currently available to provide financial assistance to MNCs but are not being used at the time of the interview. |
|  |  |  |  |  | Equipment: A collection of resources aimed at equipping neurological care providers and neurological care systems. |
|  |  |  |  |  | Infrastructure: Both physical and organizational structures and facilities that are currently in place and either support neurological care or can aid in the design and implementation of MNCs |
|  |  |  |  |  | Staffing: includes every individual who is employed in the provision of neurological care or MHC care that can be sourced for MNCs. |
|  |  |  |  |  | Supplies: Items needed to provide neurological care, such as gloves, in a hospital setting or at MNCs or MHCs that can be easily accessed by MNC operations |
|  |  | **Access to Knowledge & Information** | Accessible guidance and/or training that can be used to design, implement, and deliver the innovation. Also, knowledge and information to understand importance of healthcare and it's benefits |  |  |
| **INDIVIDUALS DOMAIN** | The roles and characteristics of individuals. | **High-level Leaders** | Individuals with significant authority, such as key decision-makers, executive leaders, or directors that will influence MNCs. |  |  |
|  |  | **Mid-level Leaders** | Individuals with a moderate level of authority, such as leaders who supervise others and are supervised by a high-level leader. |  |  |
|  |  | **Opinion Leaders** | Individuals who have an informal influence on others' attitudes and behaviors. | **Religious Leaders** |  |
|  |  |  |  | **Local leaders** | Includes Local council fives (LC5s) and other local leaders |
|  |  |  |  | **Traditional/cultural leaders** |  |
|  |  | **Implementation Facilitators** | Individuals with subject matter expertise who provide assistance, coaching, or support during implementation. Might include: Medical Officers, CHWs among other |  |  |
| **CHARACTERISTICS SUBDOMAIN** | document the MNC characteristics applicable to the roles in the MNC project. | **Roles of Providers** | Describes the providers' working conditions and experiences at the time of the interview. | **Working Experience of healthcare providers** | Providers' experience and responsibilities in the field at the time of the interviews |
|  |  |  |  | **Providers' Workflow** | a list of tasks that providers complete to provide care to patients |
|  |  | **Desire to work in MNCs** | expression of commitment and enthusiasm to work in an MNC environment | **Capability** | Provide expresses the interpersonal skills, knowledge, and competence necessary to perform the MNC/MHC related roles. |
|  |  |  |  | **Motivation** | individual expresses committed to fulfilling Role |
|  |  |  |  | **Opportunity** | Individual express the availability, scope, and power to carry out MNC related roles. |
| **IMPLEMENTATION PROCESS DOMAIN** | explains the procedures and tactics used to put the innovation into practice. Record the activities and strategies being used to implement the innovation, as well as the implementation process framework. Differentiate between the innovation (the "effects/impact" that persist after implementation is complete) and the implementation process that was used to implement it (activities that end after implementation is complete). | **Assessing Needs** | information about patients's priorities, preferences, and needs | **Considerable Disorders** | Treatable disorders that providers know can be treated in MNC settings for sure. Preventable Disorders: disorders that providers know can be prevented in MNC setting for sure |
|  |  |  |  | **Non Considerable Disorders** | disorders that providers know cannot fit MNC for sure. Not Treatable disorders: disorders that providers know cannot fit MNC for sure. |
|  |  |  |  | **Sensitization:** | Avenues of increasing awareness about Neurological care services |
|  |  |  |  | **Other Needs** | Patient Screening: Provider describes screening as a service MHC/MNC provides to the patients |
|  |  |  |  |  | Patient Follow-Up: Provider describes follow-up as a service MHC/MNC provides to the patients |
|  |  |  |  |  | Patient Medication: Medication that patients may get from MNCs/MHCs |
|  |  |  |  |  | Patient Rehabilitation: Provide explains that patients are looking for rehabilitation/rehab |
|  |  |  |  |  | Patient referral: Establishes how MNCs/MHCs can refer patients for care |
|  |  | **Assessing Context** | information to identify and assess barriers and facilitators to the innovation's implementation and delivery | **Community Level Barriers** | Where the community contributes to the circumstances that prevent patients from seeking, reaching, and receiving neurological care. |
|  |  |  |  | **Hospital Level Barriers** | Where Hospitals contribute to the circumstances that prevent patients from seeking, reaching, and receiving neurological care. |
|  |  |  |  | **Individual Level Barriers** | Individual circumstances that prevent patients from seeking, reaching, and receiving neurological care |
|  |  |  |  | **System Level Barriers** | Circumstances affecting the entire system, including the community, hospital, and patients' ability to seek, reach, and receive neurological care |
|  |  |  |  | **Resources needed** | Any other aspects that make current MNCs/MHCs/healthcare easier to implement. May include all of the resources that patients have, such as finances, family members who can be helpful when seeking neurological care, and resources that can be traded to obtain needed neurological care. Examples include: Personal assets/Belongings, Patient Support structure |
|  |  | **Planning** | The providers outline specific benchmarks that can be measured at each stage of implementation. |  |  |
|  |  | **Tailoring Strategies** | Making decisions that operationalize implementation strategies described by providers to address barriers, make use of facilitators, and fit context. |  |  |
|  |  | **Engaging** | The providers explain how to attract and promote participation in the innovation's implementation. | **Innovation Deliverers** | Constantly encourage and attract deliverers to join the implementation team and/or to deliver the innovation. |
|  |  |  |  | **Innovation Recipients** | Encourage recipients to join the implementation efforts and/or take part in the innovation. |
|  |  | **Doing** | Implement in small steps, tests, or cycles of change to trial and optimize the delivery of the innovation over time. | **Project Activities** | All recommendations where the Healthcare providers outline the steps for implementing MNCs that may be discontinued after a while.  Task Sharing: Provider explains how they share tasks with other groups/people. |
|  |  |  |  |  |  |
|  |  |  |  | **Level of Expectation** | Expectations of what MHC/MNCs should achieve from both providers and/or the general public |
|  |  | **Reflecting & Evaluating** | Providers recommend tools for gathering and discussing quantitative and qualitative data about the success of innovation and its implementation. | **Implementation Measurements** | Tools that can be customized to collect data for MNCs during implementation |
|  |  |  |  | **Innovation Measurements** | Tools that can be customized to collect data about innovation |
| **EXPERIENCE FOR MHC, NEUROLOGICAL CARE OR BOTH** | Provider's Experience for MHC, Neurological care or both | **Neurological care Experience** | provider's experience with Neurological care | **No Previous experience** |  |
|  |  | **Both Neurological care and MHC Experience** | provider's experience with both Neurological care and MHC | **Previous experience** |  |
|  |  | **MHC Experience** | provider's experience with MHC |  |  |
| **EDUCATION** | Providers describe any existing educational resources that may aid in innovation. | **Neurosurgery & Neurology Education** | Providers describe any existing neurosurgical and neurological education resources as well as future neurosurgical and neurological education resources that may aid in innovation. | **Current Education** |  |
|  |  |  |  | **Need for Education** |  |
|  |  | **MHC Education** | Providers describe any existing MHC education resources as well as future MHC education resources that may aid in innovation. |  |  |
| **CURRENT NEUROLOGICAL CARE PERCEPTIONS** | A method of comprehending neurological care; neurological care perceptions of how people in the community comprehend neurological care | **General Population** | Assumptions providers have about the general population in the rural and urban settings | **rural attitudes** | way of considering neurological care |
|  |  |  |  | **rural Beliefs** | trustworthiness of neurological care |
|  |  |  |  | **rural Knowledge** | familiarity of neurological care |
|  |  |  |  | **rural Practices** | habits surrounding neurological care |
|  |  |  |  | **urban attitudes** |  |
|  |  |  |  | **urban Beliefs** |  |
|  |  |  |  | **urban Knowledge** |  |
|  |  |  |  | **urban Practices** |  |
|  |  | **HealthCare Providers** | Assumptions providers have about the healthcare providers | **Attitudes** | way of considering neurological care |
|  |  |  | Code Attitudes, Beliefs, Knowledge, & Practices | **Beliefs** | trustworthiness of neurological care |
|  |  |  |  | **Knowledge** | familiarity of neurological care |
|  |  |  |  | **Practices** | habits surrounding neurological care |
| **CURRENT HEALTHCARE SETTING** |  | **Hospital Organization** | explains the standards the hospital employs when scheduling patients, from emergency cases to elective cases. Includes: Immediate triaging-Patients who need life-saving interventions (category 1) must be taken immediately to the resuscitation room, and emergency medicine specialists must be called at once. Urgent triaging - These patients have conditions that don't require immediate neurological or neurosurgical care. These patients may be ambulatory and self-referred, but stable. non-urgent triage - These are patients who can be scheduled to return at a later time for their elective care. And also descriptions of how the hospital is organized currently. |  |  |
|  |  |  |  |  |  |
|  |  |  |  |  |  |
|  |  | **Consultations** | explains all the consultations that healthcare providers can have at any time and anywhere. | **Neurological care consultations** | Indicate whether a consultation relates to neurological care or not. |
|  |  |  |  | **In-hospital Consultations** | inside-the-hospital department consultations |
|  |  |  |  | **Out-hospital Consultations** | consultations from non-hospital sources |
|  |  | **patients receiving care at MHCs** | The number or description of patients receiving care at MHCs according to providers |  |  |
